# Photodynamic Inactivation of Antibiotic-Resistant and Sensitive *Aeromonas hydrophila* with Peripheral Pd(II)- vs. Zn(II)-Phthalocyanines

**DOI:** 10.3390/biomedicines10020384

**Published:** 2022-02-05

**Authors:** Vanya N. Mantareva, Vesselin Kussovski, Petya Orozova, Lyudmila Dimitrova, Irem Kulu, Ivan Angelov, Mahmut Durmus, Hristo Najdenski

**Affiliations:** 1 Institute of Organic Chemistry with Centre of Phytochemistry, Bulgarian Academy of Sciences, 1113 Sofia, Bulgaria; ipangelov@gmail.com; 2 The Stephan Angeloff Institute of Microbiology, Bulgarian Academy of Sciences, 1113 Sofia, Bulgaria; vkussovski@gmail.com (V.K.); lys22@abv.bg (L.D.); hnajdenski@gmail.com (H.N.); 3 National Diagnostic Research Veterinary Institute, 1000 Sofia, Bulgaria; Nrl-fmcd@bfsa.bg; 4 Department of Chemistry, Gebze Technical University, Gebze 41400, Kocaeli, Turkey; iremkulu@gtu.edu.tr (I.K.); durmus@gtu.edu.tr (M.D.)

**Keywords:** antimicrobial multidrug resistance (AMR), photodynamic therapy (PDT), palladium and zinc phthalocyanines, antibiogram of bacterial isolates, *Aeromonas hydrophila*

## Abstract

The antimicrobial multidrug resistance (AMR) of pathogenic bacteria towards currently used antibiotics has a remarkable impact on the quality and prolongation of human lives. An effective strategy to fight AMR is the method PhotoDynamic Therapy (PDT). PDT is based on a joint action of a photosensitizer, oxygen, and light within a specific spectrum. This results in the generation of singlet oxygen and other reactive oxygen species that can inactivate the pathogenic cells without further regrowth. This study presents the efficacy of a new Pd(II)- versus Zn(II)-phthalocyanine complexes with peripheral positions of methylpyridiloxy substitution groups (pPdPc and ZnPcMe) towards Gram-negative bacteria *Aeromonas hydrophila* (*A.* *hydrophila*). Zn(II)-phthalocyanine, ZnPcMe was used as a reference compound for *in vitro* studies, bacause it is well-known with a high photodynamic inactivation ability for different pathogenic microorganisms. The studied new isolates of *A.* *hydrophila* were antibiotic-resistant (R) and sensitive (S) strains. The photoinactivation results showed a full effect with 8 µM pPdPc for S strain and with 5 µM ZnPcMe for both R and S strains. Comparison between both new isolates of *A.* *hydrophila* (S and R) suggests that the uptakes and more likely photoinactivation efficacy of the applied phthalocyanines are independent of the drug sensitivity of the studied strains.

## 1. Introduction

Among the most harmful bacterial pathogens for human health is *Aeromonas hydrophila*, with the characteristics of a foodborne pathogen of emergent status [1]. The genus *Aeromonas* is characterized as a waterborne and food-developed opportunistic Gram-negative bacteria [2]. The species has been considered as causing a wide spectrum of human diseases, such as wound infections, bacteremia and septicemia, and persistent infections in immunocompromised patients [3]. *A. hydrophila* has the ability to grow at cold temperatures, which may be a negative aspect concerning the safety of human lives. Moreover, observations showed that *A. hydrophila* causes infections in humans with a low capability for inactivation with the clinically approved treatments [4,5,6]. The scientific curiosity about this species has increased because of the following reasons: (i) the fast distribution of *Aeromonas* all over the world, (ii) the trials for the correct identification and sorting of pathogenic *Aeromonas* species, (iii) the incidence of strains with antimicrobial resistance, and (iv) the ability of some strains to remain alive after the conventional wastewater treatments [5]. Poor water quality, the ubiquitous nature and rapid spreading of harmful pathogens, environmental adverse conditions, and high stocking densities are important factors that contribute to the wide-spreading of infections [6]. These characteristics, together with the rising resistance in common pathogenic bacteria to the known drugs, are what make disease prevention a difficult mission. The determination of the natural horizontal gene transfer showed that *Aeromonas* sp. can acquire mobile genetic elements protecting antimicrobial resistance [7]. An example is the plasmid-mediated quinolone resistance (e.g., qnrS and aac (60)-Ib-cr), carbapenemase (e.g., blaKPC, blaNDM, blaGES, blaIMP, blaVIM, and blaOXA-48), and aminoglycosides-encoding genes (e.g., rmtD), typical for *Aeromonas* species [8].

Photodynamic therapy (PDT) with porphyrinoids features as a more effective alternative strategy, which is under intensive interest to keep under control the pathogenic bacterial species causing acute infections [9,10]. Lately, PDT has been well-accepted as an emergency, low-cost antimicrobial treatment for local infections without other therapeutic options [11]. After the Golden age of antibiotics, PDT method has been of scientific interest because of the fast development of AMR and the fast increase in cases with lethal outcomes due to this resistance [12]. Antimicrobial PDT has been recently well-accepted as a potential preferable choice over the traditional antibiotics because of the lack of side effects, pathogenicity reversal, and further regrowth of the pathogenic species after the treatment [13]. Studies described aPDT as a method with a lack of development of resistance with a fast response after a single application [14,15].

Phthalocyanines (Pcs) are recognized as second-generation photosensitizers for PDT after the porphyrin derivatives, which are well-accepted as appropriate for anticancer PDT [16,17]. Presently, the studies with phthalocyanine derivatives for the inactivation of pathogens are more in the experimental stage than in clinical practice [18]. Most of the known metallophthalocyanines (MPcs) have been considered for aPDT studies because of their suitable photo-physicochemical properties, such as intensive far-red or near-infrared absorption (>670 nm) and high triplet state quantum yields with a preferable singlet oxygen generation [19]. The water-soluble and cationic MPcs coordinated with different metals and semi-metals were obtained via quaternization reaction of N-attached substitution groups [16,17,18,19]. These phthalocyanines have been well-documented as proper photosensitizers for PDT applications [20].

Palladium phthalocyanine complexes with different substitution groups are known to have promising photophysical, photochemical, and photobiological properties [21,22]. The effect of an open-shell metal ion, such as palladium (Pd^2+^), in the Pc-ligand molecule leads to an increase in the triplet state and the further singlet oxygen production with relatively high quantum yields of the recently studied Pd(II)-phthalocyanine [22]. Our previous study with Pd(II)-phthalocyanine with four non-peripheral methylpyridiloxy groups (nPdPc) suggested a promising photodynamic inactivation capacity of multidrug-resistant *Staphylococcus aureus* (MRSA) but a low efficiency for *A. hydrophila* drug-resistant strain [22].

In the present study, two new isolates of Gram-negative bacterium *A. hydrophila* as antibiotic- resistant (R) and sensitive (S) strains were characterized for their susceptibility to common antibiotics (antibiograms). The photodynamic efficiency of a newly synthesized peripheral methylpyridiloxy-substituted Pd(II)-phthalocyanine (pPdPc) was studied in comparison to the well-known similar compound Zn(II)-phthalocyanine (ZnPcMe) on both *A*. *hydrophila* strains. The new palladium phthalocyanine pPdPc was tested for the uptake behavior on these pathogenic bacterial strains. In addition, the cytotoxicity studies on representative model cell lines were performed. The obtained results suggested very promising properties of the studied phthalocyanines as photosensitizers for PDT of Gram-negative *A*. *hydrophila* strains.

## 2. Material and Methods

### 2.1. Phthalocyanines

**Figure 1:**
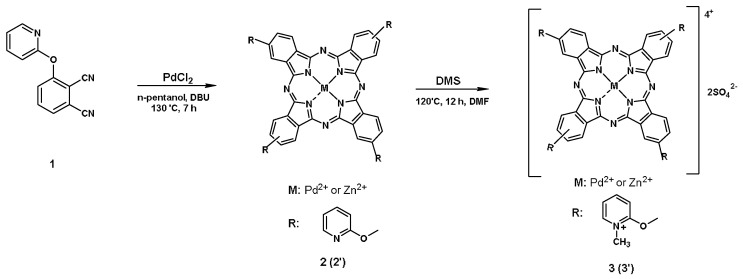
Synthesis of tetra-methylpyridiloxy-substituted Pd(II)- and Zn(II)-phthalocyanines.

The phthalocyanines of palladium (pPdPc) and zinc (ZnPcMe) were prepared and further studied as a mixture of positional isomers (Figure 1). The synthesis was carried out according to the previously published synthetic procedures which were used with slight modifications [22,23].

#### 2.1.1. Synthesis of 2,(3),9(10),16(17),23(24)-Tetrakis-[(2-pyridyloxy) phthalocyaninato] Palladium (II), (2)

A mixture of anhydrous palladium (II) chloride (0.179 g, 1 mmol), 4-pyridyloxyphthalonitrile (0.442 g, 2 mmol), DBU (3.32 mL, 2 mmol), and n-pentanol (5 mL), was stirred at 130 °C for 7 h under a nitrogen atmosphere. After cooling, the solution was dropped in n-hexane. The green solid product was precipitated and collected by filtration and washed with n-hexane. After washing, the crude product was purified by column chromatography (SiO_2_) using a CH_2_Cl_2_-MeOH (50:1) solvent system. The results were as follows: Yield: 0.86 g (52%). IR [ν_max_/cm^−1^]: 3092 (Ar-CH), 1657 (C=C), 1576, 1532, 1500, 1399, 1327, 1287, 1258, 1127, 1109 (C-O-C), 1045, 805, and 744. ^1^H-NMR (CDCl_3_): δ, ppm 7.31–7.96 (14H, m, Pc-H, and Pyridyl-H) and 6.00–6.90 (14H, m, Pc-H and Pyridyl-H). MALDI-TOF-MS *m/z*: Calc. 991.28 for C_52_H_28_N_12_O_4_Pd; Found [M]^+^ 991.38, 1014.82 [M+Na]^+^.

#### 2.1.2. Synthesis of 2,(3),9(10),16(17),13(24)-Tetrakis-{[(2-(N-methyl)pyridyloxy]phthalocyaninato} Palladium (II) Sulphate, (3)

Phthalocyanine 2 (100 mg, 0.1mmol) was heated (120 °C) in freshly distilled DMF (0.5 mL), and dimethyl sulphate (0.2 mL) was added dropwise. The mixture was stirred at 120 °C for 12 h. After this time, the mixture was cooled to room temperature, and the product was precipitated with hot acetone and collected by filtration. The green solid product was washed successively with hot ethanol, ethyl acetate, THF, chloroform, n-hexane, and diethyl ether. The resulting hygroscopic product dried over phosphorous pentoxide. The results were as follows: Yield: 0.11 g (73%). IR [ν_max_/cm^−1^]: 3064 (Ar-CH), 1655, 1578 (C=C), 1533, 1476, 1329, 1230 (S=O), 1182, 1110 (S=O), 1030 (C-O-C), 935, 837, 767, and 662 (S-O). ^1^H-NMR (DMSO-*d_6_*): δ, ppm 7.42–8.01 (28H, m, Pc-H and Pyridyl-H) and 3.86–4.44 (12H, m, CH_3_). UV/Vis (DMSO), λ_max_, nm (log ε): 318 (4.57), 623 (4.13), and 662 (4.27). MALDI-TOF-MS *m/z*: Calc. 1243.54 for C_56_H_40_N_12_O_12_S_2_Pd; Found 311.741 [(M+4)/4]^+^.

### 2.2. Bacterial Strains

Two strains of *A. hydrophila* as multidrug-resistant (R) and drug-sensitive (S) strains were recently isolated from local water resources. They were tested and confirmed as *A. hydrophila* strains by the classical microbiological tests and MALDI-TOF mass spectrometry analysis (Bruker, Munich, Germany). Trypticase soy agar (TSA) (Difco) was used for the cultivation of *A. hydrophila*. The strains were aerobically cultivated on nutrient media for 24 h at 28 °C. Cells were harvested and suspended in sterile phosphate-buffered saline (PBS) of pH 7.4. Suspensions were diluted to a cell density of ~10^6^ CFU·mL^−^^1^. The viable cells in the suspensions were counted by plating the serial dilutions on solid culture media. The number of visible colonies (colonies forming units, CFU) present on an agar plate was multiplied by the dilution factor to provide the values in CFU·mL^−^^1^.

### 2.3. Photodynamic Inactivation Study

The stock solutions of the studied phthalocyanines (~2 mM) were freshly prepared in dimethylsulphoxide (DMSO, Uvasol) and kept in a dark place during the experiments. The preparative glassware and vials were covered with aluminium foil and flushed with argon in order to prevent photobleaching of the experimental solutions. The absorption spectra were recorded on a Shimadzu UV–vis 3000 apparatus (Osaka, Japan) to control the exact concentrations before the experiments. All solids and solvents were purchased through Sigma-Aldrich (FOT, Sofia, Bulgaria).

Both strains were grown aerobically at 28 °C overnight. Then cells were harvested by centrifugation and were suspended in sterile phosphate-buffered solutions (pH 7.4). The absorbances of the cell suspensions were measured as having an optical density of 0.490 at 600 nm using a spectrophotometer Unico 2100UV, which corresponded to 10^9^ CFU·mL^−^^1^. Prior to each experiment, the cell suspensions were diluted to the bacterial density of 10^6^ CFU·mL^−1^. The cell suspensions were incubated with the tested phthalocyanine in the concentration range from 0.04 μM to 20 μM for 15 min. The portions of suspension (200 μL) were placed in a standard 96-well polystyrene plate, and light was applied. Four groups of bacterial cells were collected: (1) only cells (no light and no Pc); (2) dark control—with PS, but without light; (3) light control—without any PS, but with light irradiation; and (4) the PDI treated groups. The cells were exposed to an LED at 665 nm (ELO Ltd., Sofia, Bulgaria) with a power density of 100 mW·cm^−2^ and light dose of 50 J·cm^−2^. A portion of 0.1 mL was taken off and diluted (10-fold) with a buffer. Aliquots (0.025 mL) were spread over Trypticase^®^ Soy agar with 0.5% yeast extract. The obtained results are presented as numbers of CFU of bacteria developed for 48 h incubation (28 °C) on the agar dishes.

### 2.4. Uptake Study

In the present study, the bacterial suspensions of *A. hydrophila* as multidrug-resistant (R) and drug-sensitive (S) strains with a range of cell density (10^5^–10^8^ CFU·mL^−1^) were incubated with pPdPc with concentration 5 µM. The chemical extraction was carried out with a mixture tetrahydrofuran–sodiumdodecyl sulfate (THF: SDS, 1:1). The new phthalocyanine is water-soluble but aggregated in water media. Prior experiments, the cell suspensions were prepared by serial dilutions in phosphate buffered solutions. The applied procedure included a chemical extraction of pPdPc and fluorescence measurements (exc: 610 nm). The used protocol was previously published for non-peripheral palladium phthalocyanine, nPdPc [22].

### 2.5. Statistics

The experiments were carried out in triplicate. The collected data were presented as a mean value ± standard deviation (SD), and the difference between two means was compared by an unpaired Student’s test. *P* < 0.05 was considered as significant.

## 3. Results

Palladium and zinc phthalocyanine complexes with peripheral methylpyridiloxy substitution groups (pPdPc and ZnPcMe) were synthesized according the Refs [22,23]. The bacterial strains, *A. hydrophila,* antibiotic-resistant (R) and *A. hydrophila,* sensitive (S), were isolated and used in the photodynamic inactivation studies with Pd(II)- and Zn(II)-phthalocyanines. The antibiotic susceptibility profiles of these bacterial strains were obtained (Table 1). The results showed the following order of resistance and sensitivity: (1) the strain *A. hydrophila* (R)—resistant to Ampicillin, Ceftiofur, Florfenicol, Enrofloxacin, Co-Trimoxazol, Doxycycline, and Sparfloxacin; (2) the strain *A. hydrophila* (S)—sensitive to Ceftiofur, Florfenicol, Enrofloxacin, Co-Trimoxazol, Doxycycline, and Sparfloxacin, and resistant to Ampicillin.

**Figure 2:**
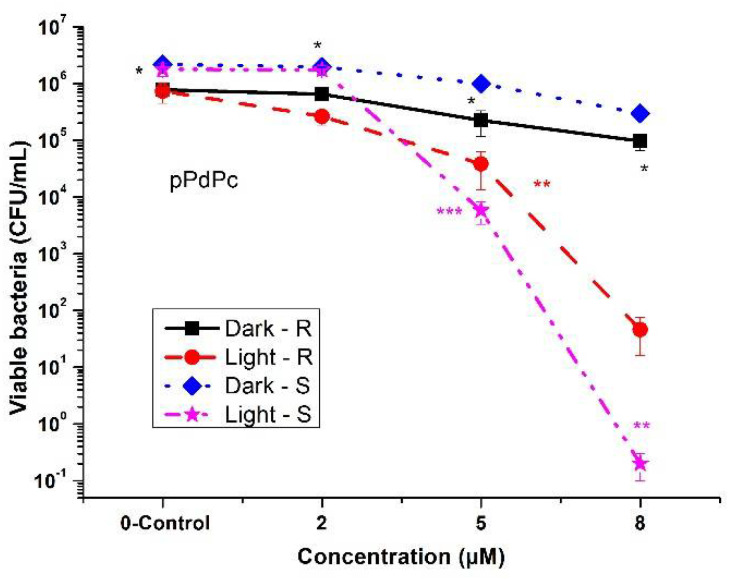
Photodynamic inactivation of *A. hydrophila* (*R* and *S*) planktonic cultured with peripheral Pd(II)-phthalocyanine (pPdPc) and an LED at 665 nm irradiation. * *p* < 0.005; ** *p* < 0.008, and *** *p* < 0.01.

Zn(II)-phthalocyanine was tested before on a number of pathogenic strains that were susceptible to the applied PDT protocol with ZnPcMe [24,25]. The peripherally substituted pPdPc, which was a new compound in this study, showed full photoinactivation ability at a concentration of 8 μM for the sensitive *A. hydrophila* strain (Figure 2). By increasing the concentrations up to 20 μM, this compound showed dark toxicity for both tested *A. hydrophila* strains (S and R). At lower concentrations (<2 μM), pPdPc showed no phototoxicity on either *A. hydrophila* S or on R strains. Comparison of the inactivation ability of pPdPc on the used *A. hydrophila* R and S strains showed log 3.32 for the R strain and resp. 5.47 log for the S strain (8 μM of pPdPc). Photodynamic inactivation with 5 μM of pPdPc was obtained with values of 2.34 log and 3.17 log toward R and S strains, respectively. In the studied concentrations, the dark toxicity was not observed. By a specific light spectrum of irradiation (665 nm), photodynamic inactivation was achieved, with slightly high results for concentrations of 5 and 8 μM for the tested antibiotic-sensitive strain of *A. hydrophila*.

**Figure 3:**
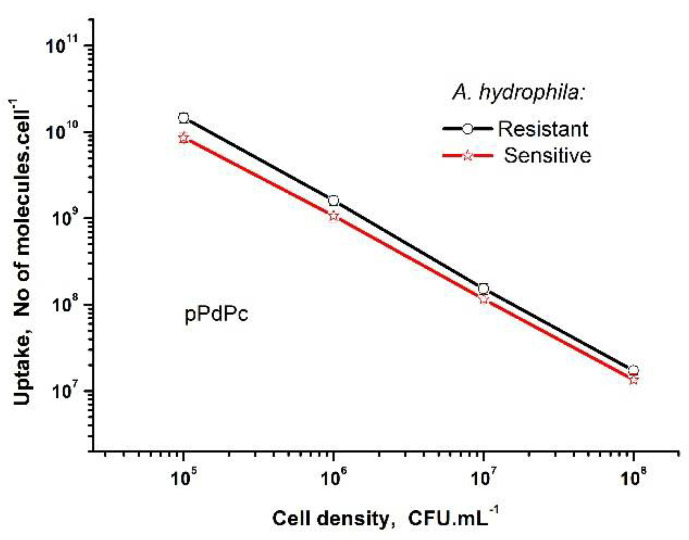
Uptake of Pd(II)-phthalocyanine (pPdPc) in *A. hydrophila* (*R* and *S*) strains as suspensions with different cell densities as obtained by fluorescence measurements.

The new cationic phthalocyanine pPdPc was evaluated for the uptake into both bacterial *A. hydrophila* strains (Figure 3). As can be seen, the uptakes showed similarity for the studied suspensions with different cell densities. The highest uptakes were obtained for the complexes incubated in more diluted suspensions in comparison to the high-density cell suspensions without the influence of the characteristics of the strain. As is well-studied, the bacterial wall of the Gram (−) species is more complicated, which additionally increases the resistance. The cationic phthalocyanines are well-studied as being more favorable for antibacterial PDT because of the proper electrostatic interaction of these compounds with cell membranes [26].

**Figure 4:**
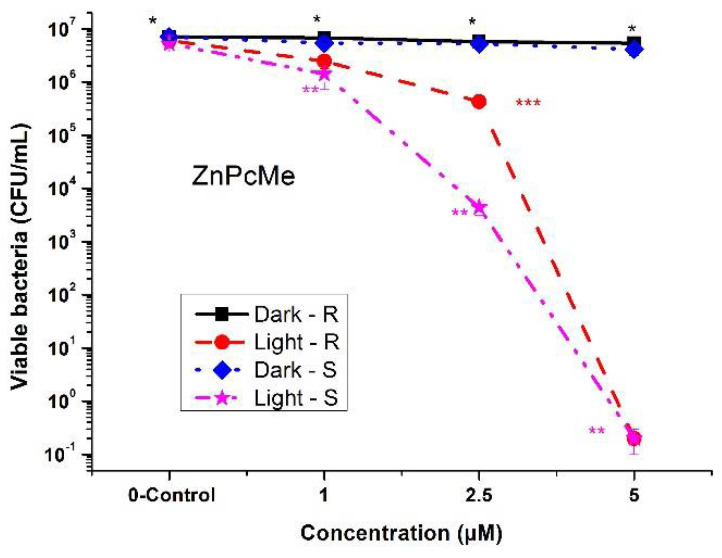
Photodynamic inactivation of *A. hydrophila* (*R* and *S*) planktonic cultured with Zn(II)-phthalocyanine (ZnPcMe) and an LED at 665 nm irradiation. * *p* < 0.005; ** *p* < 0.008, and *** *p* < 0.01.

The well-explored compound, tetra-methylpyridiloxy Zn(II)-phthalocyanine (ZnPcMe), was also tested in the present study. ZnPcMe was studied with promising photoinactivation efficacy towards pathogenic bacteria, fungus, and viruses [24,25]. For concentrations up to 5 μM, ZnPcMe was examined without dark toxicity on both tested strains (Figure 4). The full photodynamic inactivation result (~log 6) was determined for 5 μM of ZnPcMe but with low efficiency (<log 3) at lower concentrations. These observations confirm the previous studies with ZnPcMe, with photoinactivation capacity at lower concentrations [26]. Considering the structures of both peripherally substituted complexes, the phototoxic effect of ZnPcMe was higher than that of pPdPc (Figure 2 and Figure 4). However, the new pPdPc showed a strong photoinactivation ability at a concentration of 8 μM, which was higher than the concentration observed in the previous study with nPdPc on another resistant *A. hydrophila* [22]. The results demonstrated that the effect of aPDT treatment is strongly dependent on the characteristics of the strain. Additionally, in vitro studies on cell cultures were carried out and showed that Pd(II)-phthalocyanines with different positions of substituents had a lack of cytotoxicity on cell cultures, such as chick embryo fibroblasts, calf trachea cell lines, Vero cell lines, and MDBK cell lines.

## 4. Discussion

*Aeromonas* species are widely spread in water, water habitats, and in many food products, such as seafood; raw foods of animal origin, such as poultry, ground meat, and raw milk; and raw vegetables. *Aeromonas*-associated illnesses are usually caused by stress and unpredicted changes in environmental conditions. Fresh water quality, overcrowding of social communities in poor countries, undesirable changes in temperature, low oxygen and a very high amount of CO_2_ in the air, and the increase in nitrite or ammonia saturation, are considered as predisposing aspects for acute infections. *Aeromonas spp.* are known to be intrinsically resistant to many b-lactams, due to the production of multiple inducible, chromosomally encoded b-lactamases [27]. The spectrum of diseases includes gastroenteritis, septicemia, and traumatic and aquatic wound infections.

The fast development of drug resistance towards the variety of antibiotics features the emergency need of an effective approach, such as PDT, to keep harmful pathogens under control [28,29]. Previous studies to inactivate the drug-resistant *A.* *hydrophila,* which is a ubiquitous Gram-negative bacterium causing diseases in reptiles, amphibians, farm fish, and humans, showed that the drinking water isolate of this bacterium, as well as the native counterparts, are liable to PDT treatment with cationic phthalocyanine complexes, with different hydrocarbon chains as substituents [28]. The experimental photodynamic studies with *Aeromonas* sp. suggested that they are susceptible to PDT, but with different success of inactivation, in dependence on the structure of the photosensitizer and the applied irradiation conditions [22,28]. It was found that the cationic nature and optimal physicochemical properties of the phthalocyanines correlate with the uptakes and the efficiency of aPDT towards pathogenic bacteria [30,31]. Referring to the uptake of phthalocyanines by *A. hydrophila*, as was studied before, there was a noticeable inverse dependence on the cell density of suspension [28]. *A*. *hydrophila* cells incubated with Zn(II)-phthalocyanines with different lengths of hydrocarbon chains showed that an approx. 10-fold increase in cell density has resulted in a significant decrease in the uptake behavior of at least one order of magnitude [28]. The phenomenon was explained by the sterical hindrance between molecules at high bacterial density, which results in the decrease in the number of attached molecules per bacterial cell [32]. Our previous results with ZnPcMe (3 μM) showed a complete photoinactivation at a lower light dose of 30 J·cm^−2^. The advantage of the aPDT method is also presented with the current experiments with two isolates *A*. *hydrophila*, which showed significant photoinactivations (>5 log) with the applied phthalocyanines.

This study suggests that both phthalocyanines, which differ in the coordinated metal ions palladium (pPdPc) and zinc (ZnPcMe), have comparable and slightly higher photoinactivation efficacy for ZnPcMe. The full phototoxic effect was observed with complex ZnPcMe, which is known as a highly effective photosensitizer in the photodynamic inactivation of pathogenic species, including viruses [24,25]. The results with *A. hydrophila* support the role of zinc as a proper metal ion for PDT photosensitizers [26,28]. Lately, it was reported that cationic photosensitizers have a high impact on the antimicrobial efficiency because of the binding ability and the charge density distribution of cationic groups [33]. In comparison with the routine antibiotic treatment, which usually requires long-term drug usage to have a result, the PDT procedure is non-toxic to the whole human body, with a single local application of the photosensitizer and a short time of irradiation. As was reported for other pathogenic strains, the short incubation time of the photosensitizer and the mild light doses within a specific spectrum of irradiation are sufficient to kill bacteria in a single treatment, with a fast response after the procedure [34]. Meanwhile, the promising in vivo results were obtained with the Zn(II)-phthalocyanine ZnPcMe studied for the inflammatory reaction of biological tissue for applications in clinical dental practice [35,36]. These observations concluded that the photodynamic method with phthalocyanines can be considered as promising for the inactivation of pathogenic bacteria associated with acute infections in dentistry. This study showed an excellent biocompatibility with a laser at 665 nm irradiation, which was observed on experimental animals by means of histopathology [36]. In vivo examination suggested that the used in the present study ZnPcMe did not lead to damaging tissue reactions either as was compared to Fotosan^™^. This proper biocompatibility is a basis to recommend ZnPcMe for safe administration and the approval certifications for application in clinical practice.

## 5. Conclusions

The new isolates of Gram-negative bacteria *Aeromonas hydrophila* were analyzed for their susceptibility towards common antibiotics. The obtained antibiograms were for two bacterial strains of *A. hydrophila*: antibiotic-resistant (R) and sensitive (S). Both strains were used to study the photodynamic efficacy of a new palladium phthalocyanine with peripheral methylpyridiloxy groups (pPdPc). In comparison, an effective photosensitizer, such as the zinc phthalocyanine complex (ZnPcMe), was investigated. In vitro studies with the new pPdPc showed similar efficacy for both strains of *A. hydrophila* at concentrations above 5 μM by irradiation with a 665 nm light source. The novelty in the uptake study is that the bacterial cells incubated with pPdPc accumulated similar values independently on the bacterial strain—resistant (R) or sensitive (S). This indicates the ability of an analogous inactivation capacity towards the studied strains. The non-specificity of the PDT action towards both strains with similar photocytotoxicity was also shown. In this study, it was shown that the new isolates of the Gram-negative *A*. *hydrophila* species, while are challenging for inactivation with antibiotics, are susceptible to PDT with Pd(II)- and Zn(II)-phthalocyanine complexes.

## Figures and Tables

**Table 1:** Antibiotic-resistant (R) and sensitive (S) strains of *A. hydrophila*.

*Aeromonas hydrophila* (R)			*Aeromonas hydrophila* (S)		
Antimicrobial Agent	Disk Content (µg)	Sensitivity	Antimicrobial Agent	Disk Content (µg)	Sensitivity
Ampicillin	10	R	Ampicillin	10	R
Ceftiofur	30	R	Ceftiofur	30	S
Florfenicol	30	R	Florfenicol	30	S
Enrofloxacin	5	R	Enrofloxacin	5	S
Cotrimo-xazol	25	R	Cotrimo-xazol	25	S
Doxycyclin	30	R	Doxycyclin	30	S
Sparfloxacin	5	R	Sparfloxacin	5	S

## Data Availability

Not applicable.
